# Cerebellar Gray Matter Volume, Executive Function, and Insomnia: Gender Differences in Adolescents

**DOI:** 10.1038/s41598-018-37154-w

**Published:** 2019-01-29

**Authors:** Kyu-In Jung, Min-Hyeon Park, Bumhee Park, Shin-Young Kim, Yae On Kim, Bung-Nyun Kim, Subin Park, Chan-Hee Song

**Affiliations:** 1 Department of Psychiatry, St. Paul’s Hospital, College of Medicine, The Catholic University of Korea, Seoul, Republic of Korea; 2 Department of Biomedical Informatics, Ajou University School of Medicine, Suwon, Republic of Korea; 3 Department of Psychiatry and Behavioral Science, Seoul National University College of Medicine, Seoul, Republic of Korea; 4 Department of Research Planning, National Center for Mental Health, Seoul, Republic of Korea; 5 Department of Family Medicine, Yeouido St. Mary’s Hospital, College of Medicine, The Catholic University of Korea, Seoul, Republic of Korea; 6 Office of Biostatistics, Ajou Research Institute for Innovative Medicine, Ajou University Medical Center, Suwon, Republic of Korea

## Abstract

The cerebellum is an important region responsible for adolescent cognitive function and sleep, and their correlation is expected to show different patterns depending on age and gender. We examined the regional cerebellar gray matter volume (GMV), executive function (EF) and insomnia symptoms to identify their correlation and gender differences in adolescents. Data for a total of 55 subjects’ (M = 31, F = 24, 14.80 ± 1.39 years old) were analyzed. The correlations between cerebellar regional GMV and Wisconsin card sorting test (WCST) subcategories showed that EF was better with larger GMV both in males and females. Far more overall correlations with cerebellar regions were observed in boys, with corresponding correlation strength being higher, and differences in localization were also observed in contrast to girls. Larger cerebellar GMV corresponded to better EF in adolescents. Insomnia did not influence the correlations between cerebellar regional GMV and EF, but more severe insomnia in boys correlated to smaller GMV in the right flocculonodular lobe. These results might implicate that the adolescent cerebellum is involved differently in EF dependent on gender.

## Introduction

Motor function has been the main point of interest in cerebellar function^1,2^. After Leiner *et al*.^3^ suggested that the cerebellum is involved in mental skills such as learning, cognition, and language, subsequent studies^4–8^ have revealed that the cerebellum is also a major site responsible for high order cognitive processing (executive function, EF)^7,8^. The fact that the cerebellum enlarged at a slow rate during the human evolution, and that it is relatively large compared to other mammalian species supports this view^9^.

The brain region responsible for cognitive processing is not limited to a specific localization, but widely distributed in the cortical and subcortical areas^10,11^. The lateral prefrontal cortex is the critical area for EF and is associated closely with the cerebellum^12^. The cerebellum forms a corticocerebellar loop with the cortex responsible for EF. Information from the cortex is transferred to the cerebellum (especially the ventral dentate nucleus)^13,14^ via the pons, and feedback is provided the cortex via the thalamus^15^.

Studies based on neuropsychological tests^16,17^ and neuroimaging^18–21^ have shown impaired cognitive function in patients with cerebellar disability. Schmahmann and Sherman have defined cerebellar cognitive affective syndrome as a cerebellar lesion accompanied by EF, spatial cognition, personality change, and language deficit^22^.

Previous studies have suggested functional topography of the cerebellum related to various cognitive domains. Based on fMRI studies, Stoodley *et al*. have reported that the regions responsible for motor, cognition, and affect in the cerebellum are all different^23–29^. Voxel-based morphometry (VBM) studies have revealed differences in cerebellar regional gray matter volume (GMV) dependent on cognitive domains, and showed that larger GMV results in enhanced cognitive function^23–29^. Nadkarni *et al*. reported that gait speed was faster and information-processing ability was better in larger cerebellar GMV regions associated with cognitive function^30^, while Miller *et al*. observed that the GMVs of the cerebellar vermis VI, V, VIII, IX, X regions are correlated with cognitive ability^24^. In addition, the correlation between cerebellar GMV and cognitive ability shows gender difference, with males having a higher correlation strength^26^. In a nutshell, all these clinical, functional and morphological studies support the fact that the cerebellum is involved in cognitive function.

The cerebellum is also closely associated with sleep^31^. Cerebellar activity is diminished in several sleep disorders, and various sleep problems accompany cerebellar lesions^32–34^. In cerebellar ataxia, sleep disorders such as Rapid Eye Movement (REM) sleep behavior disorder, restless legs syndrome, periodic leg movement disorder, excessive daytime sleepiness, insomnia, and obstructive sleep apnea also appear frequently depending on the area of cerebellar degeneration^32,33,35–37^. Several resting-state fMRI studies in adult insomnia patients have shown decreased regional connectivity in the right anterior cerebellar lobe^38,39^. If cognitive impairment is present, default mode network (DMN) and posterior cerebellar connectivity is also disrupted^40^, suggesting that the cerebellum is closely associated with both insomnia and cognitive dysfunction. Insomnia is the most common sleep disorder in adolescents, with a lifetime prevalence of 10.7% in USA 13–16 year-olds according to Diagnostic and Statistical Manual of Mental Disorders, Fourth Edition (DSM-IV), and most of them show a trend towards becoming chronic^41,42^. This may have significant consequences on EF responsible for daily life and education in this age period^43,44^.

As frontal lobe myelination continues to 15 years of age, brain development occurs very actively in the youth period^45^. Cerebellar volume increases in an inverted U shape according to age, and the peak shows gender difference (11.8, 15.6 in female, male, respectively), with late development in boys, but on the other hand, the total volume is greater by 10–13%^46^.

EF develops fast during early and middle childhood, and slows abruptly during late childhood and adolescence^47^ and this also shows gender difference^43,47^.

According to previous studies, the cerebellum is an important region responsible for adolescent cognitive function and sleep, and their correlation is expected to show different patterns depending on age and gender. Unfortunately, most of these studies have been performed in adults. One important difference between adult and adolescent brains is cerebellar neural pruning which proceeds until puberty^48,49^. It is not yet known exactly how the cerebellum’s neural pruning progresses, but considering the fact that the cerebellar neurons account for 80% of the total brain neural cells^50^, they will be far more affected by pruning than other brain regions. The adolescent cerebellum is affected actively by neural pruning, and in regard to the cerebellar regional volume and cognitive processing, it may be significantly different from that of an adult. As far as we know, this study is the first one to look at the GMV of the cerebellum, cognitive function, and sleep problem in adolescents at the same time. With this background in mind, the present study measures the regional GMV of the cerebellum, EF, and insomnia, and attempts to identify their correlation and gender differences in adolescents.

## Results

### Demographic and clinical characteristics of the participants

The girls were younger (14.25 ± 1.03 vs 15.23 ± 1.50, P < 0.006), and showed better performance on the IQ test (P < 0.036) and total errors (TE) (P < 0.040), a subcategory of Wisconsin card sorting test (WCST), but these differences were not significant after adjusting for age (Table 1).

**Table 1:** Demographic and Clinical Data According to Gender.

Characteristics	Mean ± SD		P (Age adjusted)
	Boys (N = 31)	Girls (N = 24)	
Age	15.23 ± 1.50	14.25 ± 1.03	0.006
ISI	11.35 ± 4.90	10.33 ± 5.16	0.457 (0.436)
IQ	98.33 ± 15.14	106.19 ± 10.90	0.036 (0.130)
CDI total	15.52 ± 7.35	15.19 ± 7.64	0.858 (0.259)
**WCST**			
Total Errors (TE)	54.35 ± 59.29	59.29 ± 8.90	0.040 (0.203)
Perseverative Responses (PR)	57.71 ± 11.23	60.71 ± 8.83	0.287 (0.512)
Perseverative Errors (PE)	57.58 ± 11.00	60.50 ± 9.11	0.298 (0.620)
Nonperseverative Errors (NPE)	51.65 ± 8.08	56.79 ± 9.36	0.060 (0.141)
Conceptual Level Responses (CLR)	54.68 ± 8.78	58.79 ± 9.06	0.095 (0.208)
Categories Completed (CC)	5.61 ± 0.96	5.83 ± 0.48	0.307 (0.423)
Trials to Complete 1^st^ Category (TCF)	13.65 ± 5.12	13.29 ± 4.21	0.785 (0.367)
Failure to Maintain Set (FMS)	0.09 ± 1.38	0.67 ± 1.13	0.498 (0.580)
Learning to Learn (LL)	0.73 ± 5.68	0.13 ± 5.17	0.515 (0.638)

ISI: Insomnia Severity Index.

CDI: Children’s Depression Inventory.

WCST: Wisconsin Card Sorting Test.

### Correlation analysis of regional gray matter volume, executive function, and insomnia severity index

**Figure 1:**
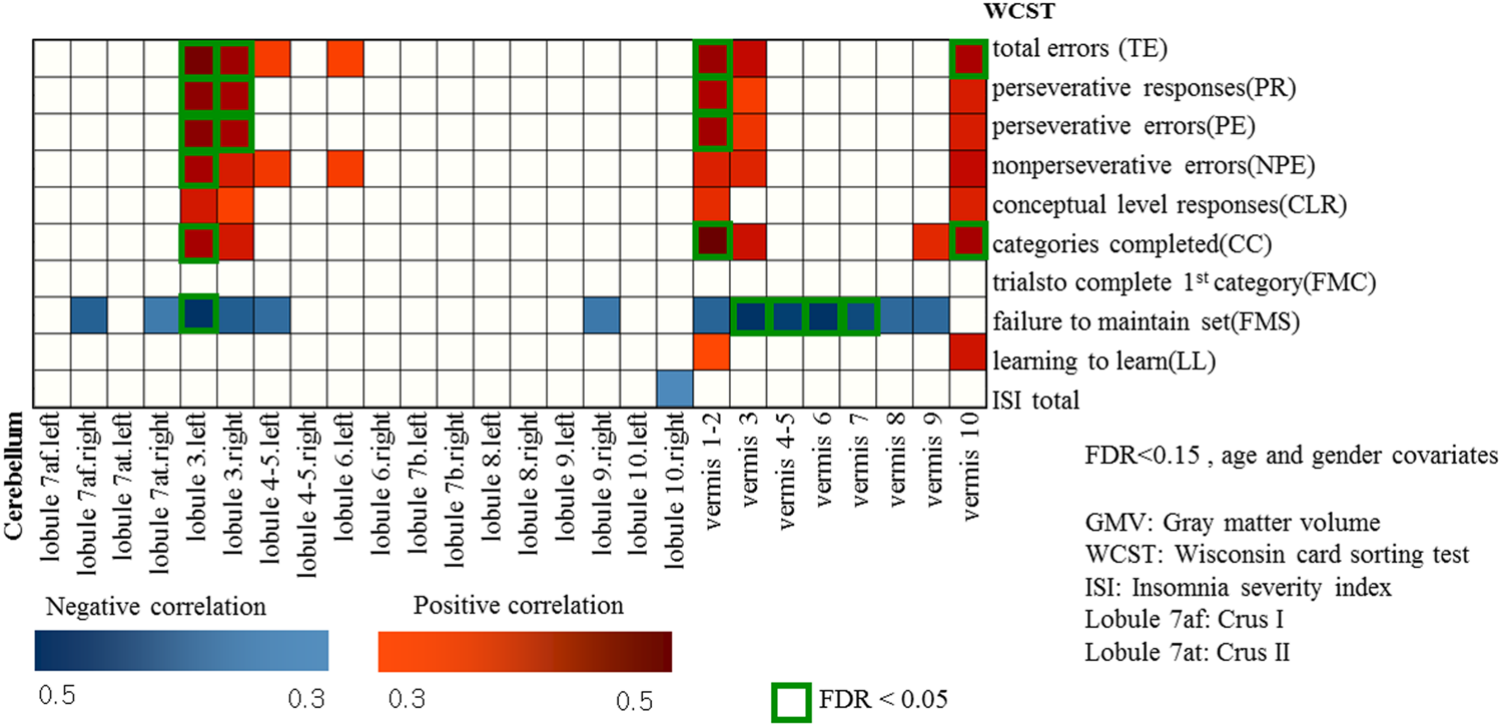
Partial correlation between cerebellar gray matter volume and Wisconsin Card Sorting Test scores (controlled for age and gender).

We determined the cerebellar regions with significant correlation (P < 0.05, controlled for age and gender) between each subcategory of WCST and GMV (Fig. 1).

**Figure 2:**
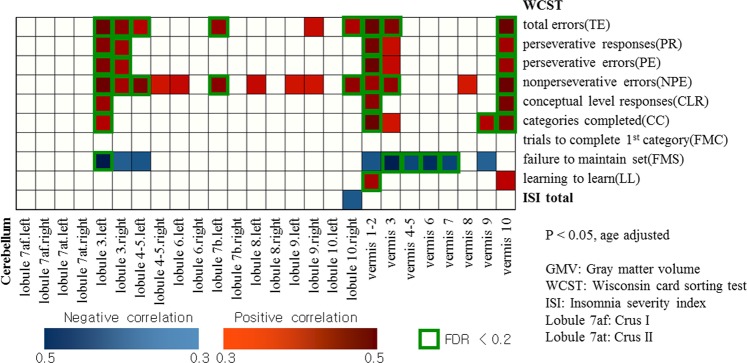
Partial correlation between cerebellar gray matter volume and Wisconsin Card Sorting Test scores in boys (controlled for age).

In boys (Fig. 2), positive correlations (controlled for age) were observed in the following: TE and lobule 3 (left, right), 4–5 (left), 7b (left), 9 (right), 10 (right), vermis 1–2, 3, 10; Perseverative Responses (PR), Perseverative Errors (PE) and lobule 3 (left, right), vermis 1–2, 3, 10; Nonperseverative Errors (NPE) and lobule 3 (left, right), 4–5 (left, right), 6 (left), 7b (left), 8 (left), 9 (left, right), 10 (right) vermis 1–2, 3, 8, 10; Conceptual Level Responses (CLR) and lobule 3 (left), vermis 1–2, 10; Categories Completed (CC) and lobule 3 (left), vermis 1–2, 3, 9, 10; Learning to Learn (LL) and vermis 1–2, 10. Failure to Maintain Set (FMS) and lobule 3 (left, right), 4–5 (left), vermis 1–2, 3, 4, 5, 6, 7, 9 showed negative correlations. No significant correlation was observed with Trials to Complete 1st Category (TCF).

**Figure 3:**
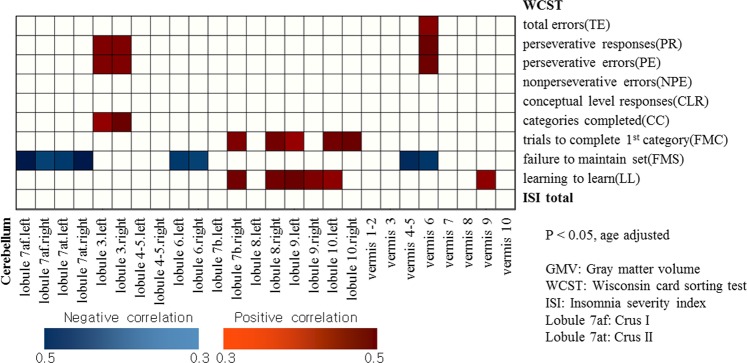
Partial correlation between cerebellar gray matter volume and Wisconsin Card Sorting Test scores in girls (controlled for age).

In girls (Fig. 3), positive correlations (controlled for age) were observed in the following: TE and vermis 6; PR, PE and lobule 3 (left, right), vermis 6; CC and lobule 3 (left, right); TCF and lobule 7b (right), 8 (right), 9 (left), 10 (left, right); LL and lobule 7b (right), 8 (right), 9 (left, right), 10 (left), vermis 9. FMS and lobule 6 (left, right), 7af (left, right), 7at (left, right), vermis 4–5, 6 showed negative correlations. No significant correlations were observed with NPE and CC.

**Figure 4:**
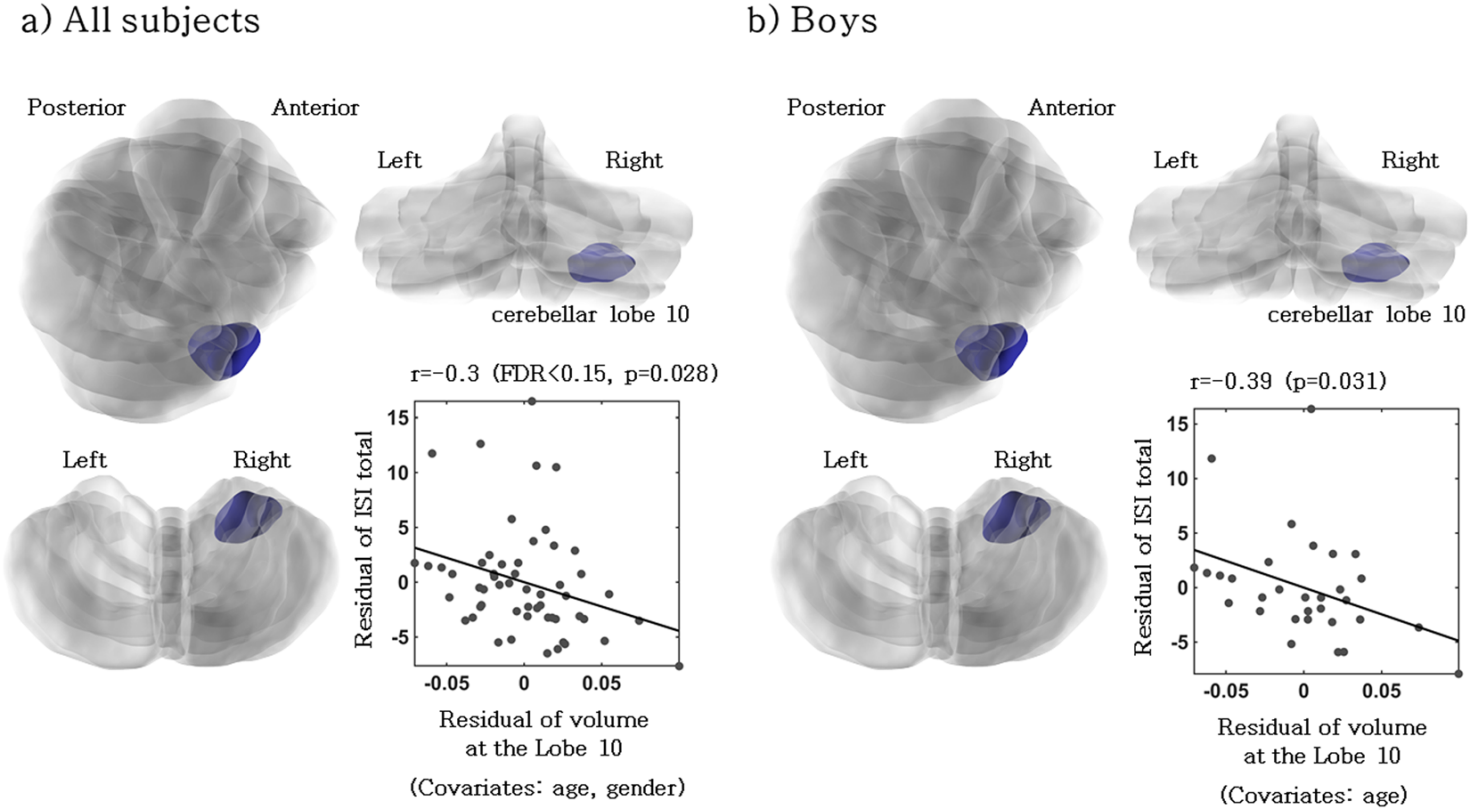
Negative correlation between the volume at the right cerebellar lobe 10 and insomnia severity scale score (controlled for age and gender/controlled for age).

ISI showed negative correlation (controlled for age and gender) with GMV of lobule 10 (right) (Figs 1 and 4) in the total participants. In boys, insomnia severity index (ISI) showed negative correlation with GMV of lobule 10 (right) (Figs 2 and 4), and negative correlation (controlled for age) with WCST subcategories CLR, LL and CC (Table 2). In contrast, no significant correlation (controlled for age and gender) was observed in regional GMV or WCST subcategories in girls (Table 2 and Fig. 3).

**Table 2:** Partial Correlation between Insomnia Severity Index and Wisconsin Card Sorting Test with Age Controlled (P < 0.05 uncorrected).

WCST subcategories	r	
	Boys (N = 31)	Girls (N = 24)
Total Errors (TE)	−0.299	−0.013
Perseverative Responses (PR)	−0.306	0.209
Perseverative Errors (PE)	−0.314	0.221
Nonperseverative Errors (NPE)	−0.275	−0.120
Conceptual Level Responses (CLR)	−0.390 (P = 0.033)	−0.061
Categories Completed (CC)	−0.392 (P = 0.032)	−0.003
Trials to Complete 1^st^ Category (TCF)	−0.046	0.033
Failure to Maintain Set (FMS)	−0.018	0.214
Learning to Learn (LL)	−0.425 (P = 0.019)	0.146

WCST: Wisconsin Card Sorting Test.

Based on previous studies showing that depressive symptoms have different effects on EF according to gender^51,52^, we reanalyzed our data after controlling for children’s depression inventory (CDI) total scores as covariates. However, our results did not change significantly in either boys or girls (Supplementary tables).

## Discussion

In the present study, the correlation strength between WCST subcategories and cerebellar GMV differed by cerebellar region. The degree of correlation also varied according to gender. Far more overall correlations with cerebellar regions were observed in boys, with corresponding correlation strength being higher, and differences in localization were also observed in contrast to girls.

The correlations between cerebellar regional GMV and WCST subcategories showed that EF was better with larger GMV. It has been previously known that the sensorimotor domain corresponds to the anterior cerebellar lobe, part of lobule 6 in the posterior cerebellar lobe, and lobule 8, while the cognitive domain corresponds to part of lobule 6 in the posterior cerebellar lobe, and lobule 7^53^. But we have found slightly different results regarding the cognition -related areas presented in these previous studies. Several factors, especially differences in EF test tool, and characteristics of participants, have to be considered when comparing our study with previous studies.

First, most previous studies have used different EF tests other than WCST, so this factor may have contributed partially to the differences observed in our study. The WCST used in this study is commonly recognized as a representative test reflecting EF and is used widely, but detailed differentiation is limited in some cases^54^. Previous studies have reported that damage to regional brain areas not considered to be directly involved in EF caused impairment of WCST performance^55^. In other words, this suggests that various regions of the brain are likely to be involved in WCST performance due to the complexity of the WCST performance itself. As a good example in explaining it, activations of primary motor lateral premotor and supplementary motor area responding to visuo-spatial cues and causing motor response and cerebellum were remarkable in a positron emission tomography (PET) study performed with WCST^56,57^. In addition, Nagahama *et al*. performed abbreviated WCST to exclude the effect of maintenance of sets, and it showed that attention to color was correlated with the activation of the ventro-occipital and left superior occipital cortex, while attention to shape was correlated with the inferior occipital cortex respectively^56^.

The GMV of the anterior cerebellum was highly correlated with the WCST subcategories in this study. Interestingly, previous functional neuroimaging studies have demonstrated prominent activation of the anterior cerebellum during WCST performance, although the anterior cerebellum is not considered to be highly involved in EF^55,56,58^.

Second, these previous studies have been done mostly in adults, while our study was done in adolescents. Although this study is cross-sectional, the fact that the different ages of the participants produce different results can be inferred indirectly by comparing the differences in development patterns of the cerebellum between boys and girls. As we mentioned earlier, the cerebellar volume changes according to age in an inverted U-shape^46,59,60^, and the cerebellum is also a part of the brain which has big differences in development depending on gender^59^. The average age of the boys participating in this study was about 15 years and it has been known that the cerebellar volume in this age reaches the peak. On the other hand, the average age of the girls participating in this study was about 14 years, and the cerebellum volume starts to decrease after 2 years of the peak period.

In addition, for girls, the relationship between the anterior and inferior lobe tends to get weaker with advancing age, but for boys, this relationship doesn’t change^46^. Among the cerebellar regions, the inferior posterior lobe is most closely related to the frontal gray matter, which is the core area of EF, and develops in a pattern similar to that of the frontal gray matter^46^. Regional differences for EF in the cerebellum might be affected by the frontal gray matter, and this may explain the reason for a different cerebellar change between girls and boys with advancing age. The age of 14–15 years is still the period for active growth of frontal gray matter, and the development potential for EF is very strong.

Furthermore, most studies on both cerebellum and cognitive function have been previously performed in adolescents with either brain disorders involving the cerebellum or cerebellar injury^61^. On the other hand, some longitudinal studies on the cerebellar volume have been performed in typically developing adolescents^46,59,60^. This means that there exists some neuroimaging evidence for typically developing cerebellum in adolescents. However, none of those studies used tools that measure EF directly. So we can only posit that certain areas of the cerebellum play a specific role in EF by integrating the results from studies on cerebellar volume of typically developing adolescents and the results from studies on EF in adolescents with cerebellar dysfunction. Therefore, the results on the role of specific areas in the cerebellum of adolescents are not definitive.

Previous studies have suggested that the vermis is connected to the limbic system and functions as a limbic domain associated with emotion control^62–67^. The characteristic feature of this study is that FMS showed significant negative correlation with GMV of vermis 1–4 in boys. Additionally, GMV of vermis 5–9, although not significant, showed negative correlation, implying that selectively it is more closely related to the vermis than the other subcategories. It is also suggested that childhood depression is associated with FMS^68^.

After correcting for CDI, only a slight, but not significant increase was observed in correlation between FMS and GMV of vermis. This study was done with a cross sectional design, so it is not possible to determine the causal relationship of depression with EF and the cerebellar GMV. However, depression does not seem to have affected the correlation between FMS and GMV of the vermis. On the other hand, in girls, the FMS showed significant correlation with vermis 4–5, 6 and Crus I, II, showing a different pattern according to region from that in boys.

ISI showed negative correlation with the GMV of right lobule 10 (flocculonodular lobe) only in boys, and among WCST subcategies, CLR, LL, and CC showed negative correlation. However, the GMV of the flocculonodular lobe did not show correlation with these 3 categories, so we suggest that the correlation between ISI and the right flocculonodular lobe is independent from EF. In girls, ISI showed no significant correlation with any cerebellar GMV or WCST subcategories. Previous studies have also shown that the cognitive functions of boys are more affected by sleep than girls^43^. Previously, Joo *et al*.^69^ have reported in a VBM study of chronic primary insomnia patients that although cerebellar gray matter deficits are observed, the relationship between sleep and clinical variables associated with cognitive disorders are not clear. The flocculonodular lobe is only known to be involved in vestibular function and influences spatial learning^70^, and so far its relationship with sleep is not known. The regional function of the cerebellum is still mainly unknown. The correlation between GMV of the right flocculonodular lobe and insomnia that is only observed in boys in the present study requires further verification.

The correlation between cerebellar regional GMV and EF showed a large gender difference. Cerebellar regions that showed significant correlation with GMV and WCST subcategories were more numerous in boys (52, 37 when FDR < 0.2, versus 28, 0 when FDR < 0.2 in girls). Additionally, in boys, the areas of correlation were mainly centered on lobule 3, vermis 1–2, 3, 10, while in girls there was no particular region, and correlation strength was also relatively weak. There is no existing study that explains the reason for this sexual dimorphism. A plausible explanation may be the aforementioned differences in cerebellar development according to gender. Considering the fact that girls show earlier brain development^71^, the pruning period of adolescence may also be chronologically different, thus resulting in functional and formative differences dependent on gender.

Another possible explanation is the influence of sex hormones. There are many estrogen receptors in the cerebellum, especially in Purkinje neurons^72,73^, and the neuroprotective or neurotrophic effect of estrogen may affect EF and cerebellar volume. In studies with postmenopausal women, women receiving estrogen showed larger GMV in the cerebellum and cognitive function was better than the control group^74–76^. Another study with 12 to 27-year old participants found that in male subjects, higher endogenous testosterone corresponded with larger cerebellar GMV. Considering the results of previous studies, it is plausible that sex hormones may have contributed to the gender differences observed in the present study^77^.

However, as everything mentioned above at present is only presumed, further neurobiological studies are required in the future, in order to identify the root causes of the gender difference influence on the correlation between the cerebellar regional GMV and EF.

Limitations in this study include the small number of subjects, the fact that the total brain volume or white matter has not been taken into account, and the problem with WCST itself as a valid EF measurement tool. Also, because this study is a cross-sectional study, it cannot explain the causal relationship between cerebellar GMV and EF, and it is also limited in that we cannot be sure that every functional change accompanies a corresponding morphologic change (or vice versa).

In a nutshell, larger cerebellar GMV corresponded to better EF in adolescents. Insomnia did not influence these correlations, but more severe insomnia in boys correlated with smaller GMV in the right flocculonodular lobe. We suggest that the adolescent cerebellum is involved differently in EF according to gender.

## Methods

### Participants

Participants were recruited from one middle school and one senior high school in Seoul, South Korea. Following approval by the school principals, the investigators visited the schools explaining the purpose of the study to the students and teachers. We also mailed letters to the parents outlining the study’s objectives, guaranteeing confidentiality, providing a contact telephone number for the principal investigator, and indicating that the parents would be informed of the results after the analyses were completed. The letter also included a statement that the parents were free to refuse to respond if they did not agree with the study’s objective.

A total of 64 students between the 7th and 11th grade (age range: 12–17 years old) volunteered to participate in this study. The Institutional Review Board (IRB) for Human Subjects at Seoul National University Hospital approved the study, and participants and their parents provided informed consent prior to enrollment. Of the 64 students who participated in the study, 9 were excluded due to incomplete responses, which resulted in a total of 55 subjects (31 boys, 24 girls). The mean age ± standard deviation (SD) of the 55 subjects was 14.80 ± 1.39 years.

### Measures

#### Wisconsin Card Sorting Test (WCST)

The computerized WCST^78^ was used to measure executive function in this study. Participants were required to match response cards to the four stimulus cards along three dimensions (color, form, and number). The program calculated T scores (M = 50, SD = 10) for Total Errors (TE), Perseverative Responses (PR), Perseverative Errors (PE), Nonperseverative Errors (NPE), and Conceptual Level Responses (CLR) and raw scores for Categories Completed (CC), Trials to Complete 1st Category (TCF), Failure to Maintain Set (FMS), and Learning to Learn (LL). TE measured the total number of perseverative and non-perseverative errors. PR indicated the number of responses that were perseverative, regardless of the feedback. PE measured the inability to change reaction, indicating a tendency towards perseveration. NPE reflected the number of errors that were not perseverative. CLR was the proportion of continuously correct reactions in more than three times. CC was the number of times 10 correct responses consecutively were made. Trials to 1st Category represented the total number of trials needed to complete the first category successfully. FMS was the number of times participants made an error after 5–9 correct responses consecutively, indicating an efficiency of sorting. LL is mean changes in conceptual efficiency across consecutive categories. With FMS, lower scores reflect higher function of each subscale, whereas with the other categories including the subscales calculated by T scores, higher scores indicate higher function of each subscale.

#### Insomnia Severity Index (ISI)

The ISI was developed by Morin and colleagues^78^ and has been used for the last 20 years. It is a 7-item scale assessing the perceived severity of insomnia symptom (initial, middle, and terminal), the degree of satisfaction with sleep, the interference with daytime functioning, the awareness of impairment, and the concern caused by the sleep problems^79^. The respondents use a 5-point Likert-type scale ranging from 0 to 4 to describe their insomnia over the past 2 weeks.

#### The Children’s Depression Inventory (CDI)

The CDI is a scale that has been modified from Beck’s depression inventory for children, and translated into Korean^80^. The CDI evaluates the level of depression by self-report from children and adolescents 7 to 17 years old. It consists of 27 items, and each item is rated from 0 to 2 points according to severity. The total score ranges from 0 to 54 points, and a higher score implies a more severe level of depression^81^.

#### Magnetic Resonance Imaging (MRI) data acquistion

MRI images were acquired using a 3.0 Tesla MRI scanner (Siemens, Magnetom Tim-Trio). High-resolution T1-weighted images were obtained from each participant using a magnetization-prepared rapid acquisition gradient echo (MPRAGE) pulse sequence (repetition time (TR) = 1900 ms; echo time (TE) = 3.13 ms; flip angle = 9°; matrix size = 256 × 256; Field-of-view (FOV) = 230 × 230 mm^2^; thickness = 0.9 mm). Head motion-related artifacts during scanning were minimized by using foam pads.

#### Regional Voxel-Based Morphometry (VBM) Analysis

We first inspected all T1-weighted images visually for motion and/or other artifacts and did not find any abnormality. All T1-weighted images were spatially preprocessed using canonical VBM-DARTEL procedure implemented in statistical parametric mapping (SPM12, http://www.fil.ion.ucl.ac.uk/spm/, Wellcome Trust Centre for Neuroimaging, London, UK)^82^. The procedure includes the following steps: (1) manual reorientation to place the anterior commissure at the origin and the anterior-posterior commissure in the horizontal plane, (2) gray matter segmentation using the SPM standard tissue probability map, (3) creation of a study-specific GM template from all the images using the DARTEL procedure, (4) spatial normalization of the individual GM images using DARTEL registration and modulation using the Jacobian determinant to adjust for nonlinear differences in individual brain size, and (5) spatial smoothing with Gaussian kernel of 8 mm full-width at half maximum (FWHM). After preprocessing, regional GMV values at the cerebellum were extracted by averaging the values within 26 cerebellar sites (8 vermis regions and 9 lobule regions in each hemisphere), which are defined by automated anatomical labeling^83^.

### Statistical analysis

We performed partial correlation analyses between regional GMV in the cerebellum and several clinical scores representing WCST and ISI for boys and girls separately (covariates; age, CDI).

Levels of statistical significance were decided as false discovery rate (FDR) < 0.2 and FDR < 0.05, for controlling multiple correlation comparisons which were calculated among multiple regions and clinical variables. FDR levels of the range 0.1–0.2 are generally known to be acceptable for multiple comparison correction^84^. All statistical analyses were performed using MATLAB-based custom software (Mathworks, Sherborn, MA, USA).

### Human and Animal Rights

All procedures performed in studies involving human participants were in accordance with the ethical standards of the institutional and/or national research committee and with the 1964 Declaration of Helsinki and its later amendments or comparable ethical standards.

### Ethics Approval and Consent to Participate

The Institutional Review Board (IRB) for Human Subjects at Seoul National University Hospital approved the study (No. C-1412-081-633), and participants and their parents provided informed consent prior to enrolment.

## Supplementary information

Supplementary tables
