# Supplementary material for: Cerebellar Gray Matter Volume, Executive Function, and Insomnia: Gender Differences in Adolescents
Source: Sci Rep. 2019 Jan 29;9:855. doi: 10.1038/s41598-018-37154-w (PMC6351545; doi:10.1038/s41598-018-37154-w)
Supplement: Supplementary file 1 — Supplementary tables [file 41598_2018_37154_MOESM1_ESM.pdf]

Cerebellar Gray Matter Volume, Executive Function, and Insomnia: Gender Differences in Adolescents

Kyu-In Jung, MD, PhD[1], Min-Hyeon Park, MD, PhD[*, 1], Bumhee Park, PhD[*, 2], Shin-Young Kim, MA[1], Yae On Kim, MA[1], Bung-Nyun Kim, MD, PhD[3], Subin Park, MD, PhD[4] and Chan-Hee Song, MD, PhD[5]

[1]Department of Psychiatry, St. Paul's Hospital, College of Medicine, The Catholic University of Korea, Seoul, Republic of Korea

[2]Department of Biomedical Informatics, Ajou University School of Medicine, Suwon, Republic of Korea

[3]Department of Psychiatry and Behavioral Science, Seoul National University College of Medicine, Seoul, Republic of Korea

[4] Department of Research Planning, National Center for Mental Health, Seoul, Republic of Korea

[5]Department of Family Medicine, Yeouido St. Mary's Hospital, College of Medicine, The Catholic University of Korea, Seoul, Republic of Korea

***Co-corresponding author**

***Correspondence to:**

Min-Hyeon Park, MD, PhD

Department of Psychiatry, St. Paul’s Hospital, College of Medicine, The Catholic University of Korea, 180, Wangsan-ro, Dongdaemun-gu, Seoul, 02559, Republic of Korea Tel: +82-2-958-2153, Fax: +82-31-847-3630, E-mail: neominnie00@hanmail.net

Bumhee Park, PhD

Department of Biomedical Informatics, Ajou University School of Medicine, Worldcup-ro, Yeongtong-gu, Suwon 16499, Republic of Korea Tel: +82-31-219-4458, Fax: +82-31-219-4472, E-mail: bhpark@ajou.ac.kr

Supplementary Table 1. Partial correlation between cerebellar gray matter volume and Wisconsin Card Sorting Test scores with age and gender covariates.

	TE	PR	PE	NPE	CLR	CC	FMC	FMS	LL	ISI
Lobule 7 af.left	.170	.131	.151	.151	.135	.163	.026	-.279*	.174	-.152
Lobule 7 af.right	.147	.106	.133	.122	.093	.155	.080	-.370**	.077	-.140
Lobule 7 at.left	.146	.097	.108	.153	.083	.034	.091	-.290*	.000	-.031
Lobule 7 at.right	.099	.049	.063	.107	.020	-.033	.122	-.326*	-.063	-.069
Lobule 3.left	.483***	.451***	.458***	.423**	.368**	.421**	-.050	-.453***	.209	-.045
Lobule 3.right	.430**	.425**	.423**	.365**	.316*	.366**	-.063	-.376**	.202	-.104
Lobule 4-5.left	.321*	.234	.236	.327*	.222	.227	-.039	-.354**	.126	-.003
Lobule 4-5.right	.293*	.235	.231	.293*	.206	.206	-.052	-.287*	.152	-.043
Lobule 6.left	.316*	.234	.241	.321*	.261	.230	-.031	-.224	.210	-.132
Lobule 6.right	.246	.173	.178	.247	.185	.193	-.060	-.250	.140	-.122
Lobule 7b.left	.218	.174	.166	.230	.172	.110	.107	-.254	.090	-.152
Lobule 7b.right	.164	.128	.129	.159	.113	.074	.134	-.269	.064	-.158
Lobule 8.left	.177	.119	.103	.202	.148	.109	.078	-.183	.147	-.165
Lobule 8.right	.187	.117	.103	.207	.144	.124	.104	-.248	.131	-.205
Lobule 9.left	.208	.156	.142	.214	.140	.230	.082	-.262	.246	-.077
Lobule 9.right	.272*	.233	.222	.258	.189	.287*	.097	-.334*	.242	-.153
Lobule 10.left	.134	.154	.133	.125	.116	.133	.088	-.114	.287*	-.140
Lobule 10.right	.267	.238	.223	.275*	.240	.132	-.037	-.176	.071	-.302*
Vermis 1-2	.438**	.412**	.430**	.359**	.342*	.509***	-.002	-.368**	.306*	-.095
Vermis 3	.386**	.321*	.330*	.353**	.257	.378**	-.113	-.452***	.134	-.025
Vermis 4-5	.265	.185	.175	.273*	.137	.169	-.120	-.433**	.053	-.018
Vermis 6	.264	.180	.189	.258	.141	.229	.101	-.453***	.127	.051
Vermis 7	.142	.056	.063	.162	.042	.083	.043	-.412**	.006	.024
Vermis 8	.257	.190	.176	.270	.185	.267	.004	-.357**	.200	-.168
Vermis 9	.284*	.241	.229	.267	.209	.347*	-.002	-.359**	.267	-.109
Vermis 10	.418**	.362**	.362**	.387**	.358**	.423**	.091	-.299*	.371**	-.227

* P < .05, ** P< .01, ***P < .001

TE= Total errors, PR= Perseverative responses, PE= Perseverative errors, NPE= Non-perseverative errors, CLR= Conceptual level responses, CC= Categories completed, FMC= Trials to complete first category, FMS= Failure to maintain set, LL= Learning to learn, ISI= Total score of the Insomnia Severity Index.

Supplementary Table 2. Partial correlation between cerebellar gray matter volume and Wisconsin Card Sorting Test scores with age, gender and CDI score covariates.

	TE	PR	PE	NPE	CLR	CC	FMC	FMS	LL	ISI
Lobule 7 af.left	.166	.166	.166	.166	.166	.166	.166	.166	.166	.166
Lobule 7 af.right	.139	.079	.105	.123	.083	.119	.024	-.351*	.051	-.077
Lobule 7 at.left	.140	.080	.090	.153	.076	.008	.058	-.275*	-.018	.012
Lobule 7 at.right	.093	.033	.046	.106	.013	-.057	.093	-.313*	-.080	-.033
Lobule 3.left	.481***	.445***	.451***	.424**	.365**	.412**	-.076	-.445***	.200	-.017
Lobule 3.right	.428**	.416**	.413**	.367**	.312*	.351*	-.098	-.364**	.189	-.070
Lobule 4-5.left	.319*	.227	.229	.327*	.220	.218	-.057	-.348*	.118	.018
Lobule 4-5.right	.290*	.225	.221	.293*	.202	.192	-.078	-.276*	.142	-.015
Lobule 6.left	.313*	.223	.229	.322*	.257	.214	-.061	-.211	.199	-.103
Lobule 6.right	.242	.160	.163	.248	.180	.174	-.094	-.235	.126	-.089
Lobule 7b.left	.214	.165	.156	.230	.168	.097	.089	-.244	.081	-.133
Lobule 7b.right	.160	.119	.119	.158	.109	.061	.117	-.261	.055	-.140
Lobule 8.left	.178	.121	.105	.202	.149	.112	.083	-.187	.149	-.174
Lobule 8.right	.186	.115	.101	.207	.143	.121	.101	-.247	.129	-.205
Lobule 9.left	.210	.161	.148	.215	.142	.238	.091	-.269	.252	-.089
Lobule 9.right	.270	.228	.216	.258	.186	.281*	.085	-.329*	.237	-.141
Lobule 10.left	.139	.166	.146	.126	.121	.151	.112	-.128	.300*	-.171
Lobule 10.right	.263	.230	.213	.275*	.237	.117	-.061	-.164	.061	-.286*
Vermis 1-2	0.436**	.409**	.427**	.359**	.340*	.507***	-.014	-.364**	.302*	-.083
Vermis 3	.384**	.318*	.327*	.352*	.255	.376**	-.126	-.450***	.130	-.014
Vermis 4-5	.267	.189	.179	.273	.139	.175	-.118	-.440**	.056	-.024
Vermis 6	.261	.173	.182	.257	.138	.221	.088	-.449***	.121	.072
Vermis 7	.137	.041	.047	.162	.035	.061	.012	-.401**	-.010	.064
Vermis 8	.255	.184	.170	.270	.183	.261	-.009	-.353*	.195	-.158
Vermis 9	.287*	.247	.236	.268	.211	.358**	.006	-.368**	.273	-.123
Vermis 10	.416**	.353*	.353*	.388**	.355**	.412**	.067	-.287*	.363**	-.204

* P < .05, ** P< .01, ***P < .001

TE= Total errors, PR= Perseverative responses, PE= Perseverative errors, NPE= Non-perseverative errors, CLR= Conceptual level responses, CC= Categories completed, FMC= Trials to complete first category, FMS= Failure to maintain set, LL= Learning to learn, ISI= Total score of the Insomnia Severity Index.

Supplementary Table 3. Partial correlation between cerebellar gray matter volume and Wisconsin Card Sorting Test scores in males with age covariates.

	TE	PR	PE	NPE	CLR	CC	FMC	FMS	LL	ISI
Lobule 7 af.left	.198	.084	.102	.232	.201	.204	.194	-.158	.204	-.276
Lobule 7 af.right	.174	.120	.145	.156	.130	.193	.223	-.340	.082	-.175
Lobule 7 at.left	.255	.148	.154	.263	.176	.093	.063	-.187	-.139	-.018
Lobule 7 at.right	.130	.020	.031	.160	.009	-.080	.028	-.212	-.292	-.008
Lobule 3.left	.532**	.461*	.466**	.508**	.434*	.407*	-.079	-.508**	.220	-.080
Lobule 3.right	.457*	.428*	.421*	.424*	.358	.315	-.090	-.377*	.198	-.080
Lobule 4-5.left	.401*	.220	.226	.473**	.300	.239	.008	-.391*	.130	-.033
Lobule 4-5.right	.325	.210	.208	.371*	.241	.182	-.019	-.272	.141	-.038
Lobule 6.left	.332	.183	.191	.387*	.309	.268	.122	-.119	.264	-.183
Lobule 6.right	.261	.123	.129	.314	.212	.205	.099	-.174	.171	-.131
Lobule 7b.left	.443*	.322	.308	.456*	.352	.185	-.125	-.190	-.057	-.171
Lobule 7b.right	.289	.155	.154	.311	.186	.057	-.133	-.169	-.181	-.134
Lobule 8.left	.346	.201	.178	.386*	.282	.171	-.164	-.055	.026	-.144
Lobule 8.right	.316	.162	.143	.352	.227	.150	-.201	-.166	-.058	-.181
Lobule 9.left	.357	.225	.220	.374*	.251	.296	-.111	-.234	.145	-.002
Lobule 9.right	.382*	.292	.287	.370*	.275	.334	-.075	-.311	.159	-.102
Lobule 10.left	.310	.239	.218	.317	.270	.199	-.166	-.148	.222	-.300
Lobule 10.right	.410*	.314	.297	.447*	.360	.139	-.348	-.233	.008	-.387*
Vermis 1-2	.507**	.480**	.499**	.434*	.454*	.530**	.100	-.394*	.424*	-.044
Vermis 3	.463**	.380*	.388*	.448*	.361	.371*	-.090	-.485**	.172	-.019
Vermis 4-5	.260	.124	.106	.315	.117	.161	-.065	-.417*	-.006	-.010
Vermis 6	.182	.055	.067	.212	.039	.217	.194	-.462*	.091	.109
Vermis 7	.141	.018	.036	.162	.006	.122	.127	-.421*	-.078	.124
Vermis 8	.354	.226	.221	.370*	.272	.358	-.046	-.338	.150	-.148
Vermis 9	.351	.272	.269	.324	.264	.409*	-.120	-.371*	.215	-.069
Vermis 10	.521**	.425*	.433*	.498**	.480**	.461*	.000	-.279	.396*	-.188

* P < .05, ** P< .01, ***P < .001

TE= Total errors, PR= Perseverative responses, PE= Perseverative errors, NPE= Non-perseverative errors, CLR= Conceptual level responses, CC= Categories completed, FMC= Trials to complete first category, FMS= Failure to maintain set, LL= Learning to learn, ISI= Total score of the Insomnia Severity Index.

Supplementary Table 4. Partial correlation between cerebellar gray matter volume and Wisconsin Card Sorting Test scores in males with age and CDI score covariates.

	TE	PR	PE	NPE	CLR	CC	FMC	FMS	LL	ISI
Lobule 7 af.left	.157	.017	.031	.206	.154	.161	.145	-.147	.180	-.220
Lobule 7 af.right	.090	-.015	.003	.100	.026	.108	.128	-.345	.027	-.039
Lobule 7 at.left	.190	.033	.031	.223	.090	.005	-.041	-.174	-.209	.125
Lobule 7 at.right	.065	-.087	-.082	.118	-.076	-.165	-.060	-.201	-.356	.108
Lobule 3.left	.514**	.434*	.439*	.495**	.409*	.383*	-.131	-.505**	.201	-.024
Lobule 3.right	.430*	.388*	.379*	.405*	.321	.278	-.156	-.372*	.174	-.008
Lobule 4-5.left	.380*	.181	.185	.459*	.271	.210	-.036	-.386*	.111	.022
Lobule 4-5.right	.293	.158	.152	.351	.200	.142	-.076	-.264	.117	.032
Lobule 6.left	.291	.108	.112	.363	.259	.220	.056	-.104	.239	-.103
Lobule 6.right	.212	.038	.038	.285	.152	.150	.027	-.161	.140	-.041
Lobule 7b.left	.405*	.252	.231	.434*	.300	.125	-.220	-.178	-.103	-.080
Lobule 7b.right	.245	.079	.072	.283	.127	-.005	-.218	-.156	-.228	-.050
Lobule 8.left	.336	.182	.158	.378*	.269	.155	-.195	-.049	.014	-.121
Lobule 8.right	.298	.130	.107	.339	.203	.125	-.244	-.158	-.076	-.149
Lobule 9.left	.363	.232	.228	.377*	.256	.301	-.119	-.234	.144	.003
Lobule 9.right	.366	.267	.261	.357	.253	.316	-.113	-.305	.145	-.064
Lobule 10.left	.335	.274	.256	.333	.298	.221	-.153	-.154	.234	-.343
Lobule 10.right	.395*	.291	.272	.436*	.341	.113	-.398	-.227	-.009	-.369
Vermis 1-2	.490**	.459*	.479**	.419*	.433*	.513**	.061	-.389*	.411*	.009
Vermis 3	.451*	.364	.372*	.438*	.345	.356	-.125	-.482**	.159	.021
Vermis 4-5	.260	.121	.102	.314	.113	.158	-.076	-.416*	-.011	.000
Vermis 6	.154	.008	.016	.192	.000	.189	.161	-.458*	.072	.171
Vermis 7	.089	-.067	-.053	.128	-.062	.067	.065	-.419*	-.117	.229
Vermis 8	.332	.189	.181	.354	.243	.335	-.092	-.332	.132	-.103
Vermis 9	.358	.284	.283	.327	.271	.418*	-.126	-.372*	.216	-.071
Vermis 10	.501**	.393*	.400*	.483**	.455*	.438*	-.051	-.272	.381*	-.136

* P < .05, ** P< .01, ***P < .001

TE= Total errors, PR= Perseverative responses, PE= Perseverative errors, NPE= Non-perseverative errors, CLR= Conceptual level responses, CC= Categories completed, FMC= Trials to complete first category, FMS= Failure to maintain set, LL= Learning to learn, ISI= Total score of the Insomnia Severity Index.

Supplementary Table 5. Partial correlation between cerebellar gray matter volume and Wisconsin Card Sorting Test scores in females with age covariates.

	TE	PR	PE	NPE	CLR	CC	FMC	FMS	LL	ISI
Lobule 7 af.left	.124	.219	.239	.031	.034	.091	-.240	-.509*	.115	.031
Lobule 7 af.right	.100	.093	.124	.067	.046	.002	-.218	-.428*	.049	-.102
Lobule 7 at.left	.038	.024	.047	.054	-.016	-.012	.183	-.443*	.249	-.034
Lobule 7 at.right	.062	.082	.102	.048	.024	.130	.305	-.510*	.411	-.132
Lobule 3.left	.404	.472*	.474*	.288	.290	.445*	-.070	-.336	.152	-.001
Lobule 3.right	.397	.472*	.472*	.275	.283	.516*	-.104	-.383	.185	-.179
Lobule 4-5.left	.191	.279	.269	.112	.120	.185	-.161	-.283	.105	.032
Lobule 4-5.right	.237	.307	.298	.158	.155	.314	-.143	-.329	.188	-.057
Lobule 6.left	.295	.332	.331	.229	.183	.196	-.269	-.441*	.093	-.048
Lobule 6.right	.229	.274	.276	.147	.136	.243	-.331	-.426*	.077	-.103
Lobule 7b.left	.015	.012	.019	.045	.015	.063	.382	-.349	.340	-.134
Lobule 7b.right	.040	.094	.099	.019	.039	.177	.472*	-.412	.487*	-.178
Lobule 8.left	-.001	.009	.007	.027	.013	.062	.394	-.355	.390	-.181
Lobule 8.right	.046	.058	.054	.062	.058	.132	.490*	-.368	.486*	-.227
Lobule 9.left	-.038	.012	-.008	-.022	-.030	.089	.442*	-.317	.523*	-.184
Lobule 9.right	.077	.112	.093	.082	.057	.156	.412	-.380	.471*	-.239
Lobule 10.left	-.138	.004	-.011	-.143	-.096	-.044	.485*	-.055	.453*	.072
Lobule 10.right	-.017	.061	.059	-.028	.036	.106	.603**	-.044	.263	-.170
Vermis 1-2	.313	.285	.303	.239	.173	.382	-.324	-.315	-.110	-.221
Vermis 3	.236	.214	.230	.186	.091	.326	-.306	-.388	-.048	-.065
Vermis 4-5	.277	.349	.336	.211	.191	.137	-.308	-.467*	.185	-.046
Vermis 6	.460*	.507*	.495*	.373	.352	.333	-.073	-.448*	.264	-.050
Vermis 7	.154	.124	.108	.176	.093	.029	-.059	-.406	.278	-.129
Vermis 8	.109	.094	.079	.138	.046	.147	.177	-.411	.380	-.186
Vermis 9	.158	.155	.132	.180	.111	.190	.288	-.340	.450*	-.180
Vermis 10	.238	.249	.236	.215	.183	.300	.218	-.337	.299	-.309

* P < .05, ** P< .01, ***P < .001

TE= Total errors, PR= Perseverative responses, PE= Perseverative errors, NPE= Non-perseverative errors, CLR= Conceptual level responses, CC= Categories completed, FMC= Trials to complete first category, FMS= Failure to maintain set, LL= Learning to learn, ISI= Total score of the Insomnia Severity Index.

Supplementary Table 6. Partial correlation between cerebellar gray matter volume and Wisconsin Card Sorting Test scores in females with age and CDI score covariates.

	TE	PR	PE	NPE	CLR	CC	FMC	FMS	LL	ISI
Lobule 7 af.left	.095	.192	.213	.003	.001	.075	-.231	-.565**	.118	.006
Lobule 7 af.right	.089	.081	.113	.057	.034	-.005	-.214	-.456*	.050	-.114
Lobule 7 at.left	.030	.015	.038	.047	-.026	-.017	.187	-.467*	.250	-.042
Lobule 7 at.right	.049	.069	.089	.035	.009	.123	.313	-.542**	.414	-.146
Lobule 3.left	.398	.468*	.471*	.279	.280	.441*	-.064	-.367	.154	-.015
Lobule 3.right	.391	.468*	.468*	.265	.272	.512*	-.098	-.416	.187	-.197
Lobule 4-5.left	.175	.266	.255	.096	.102	.176	-.154	-.316	.107	.016
Lobule 4-5.right	.218	.289	.280	.138	.132	.304	-.134	-.371	.191	-.079
Lobule 6.left	.279	.317	.316	.212	.163	.186	-.263	-.484*	.095	-.069
Lobule 6.right	.211	.258	.259	.129	.115	.234	-.325	-.468*	.079	-.124
Lobule 7b.left	-.005	-.010	-.003	.028	-.006	.053	.392	-.383	.343	-.153
Lobule 7b.right	.022	.077	.082	.002	.021	.168	.483*	-.446*	.490*	-.196
Lobule 8.left	-.028	-.020	-.022	.003	-.015	.049	.408	-.398	.395	-.206
Lobule 8.right	.028	.039	.035	.047	.040	.124	.500*	-.401	.489*	-.246
Lobule 9.left	-.065	-.015	-.036	-.045	-.057	.077	.456*	-.355	.529*	-.208
Lobule 9.right	.064	.100	.080	.071	.043	.150	.420	-.408	.473*	-.255
Lobule 10.left	-.178	-.033	-.049	-.179	-.134	-.062	.505*	-.094	.460*	.048
Lobule 10.right	-.007	.074	.073	-.019	.048	.113	.602**	-.034	.262	-.165
Vermis 1-2	.273	.241	.260	.200	.124	.367	-.314	-.396	-.109	-.276
Vermis 3	.198	.173	.189	.150	.045	.310	-.296	-.462*	-.045	-.106
Vermis 4-5	.248	.321	.307	.182	.157	.119	-.298	-.534*	.191	-.080
Vermis 6	.451*	.499*	.487*	.361	.339	.325	-.064	-.490*	.267	-.070
Vermis 7	.149	.118	.101	.172	.086	.025	-.056	-.429*	.279	-.139
Vermis 8	.100	.085	.068	.130	.036	.142	.182	-.437*	.382	-.199
Vermis 9	.135	.131	.106	.160	.086	.178	.302	-.384	.456*	-.206
Vermis 10	.238	.250	.236	.214	.181	.299	.222	-.355	.299	-.319

* $P < .05$, ** $P < .01$, *** $P < .001$

TE= Total errors, PR= Perseverative responses, PE= Perseverative errors, NPE= Non-perseverative errors, CLR= Conceptual level responses, CC= Categories completed, FMC= Trials to complete first category, FMS= Failure to maintain set, LL= Learning to learn, ISI= Total score of the Insomnia Severity Index.
